# Barriers to timely identification of bipolar disorder in youth: a multidimensional perspective

**DOI:** 10.3389/frcha.2023.1186722

**Published:** 2023-05-15

**Authors:** Kamyar Keramatian, Emma Morton

**Affiliations:** Department of Psychiatry, University of British Columbia, Vancouver, BC, Canada

**Keywords:** bipolar disorder, youth, early intervention, delayed diagnosis, conceptual framework

## Abstract

Bipolar disorder (BD) in youth often goes unrecognized and therefore untreated. However, little is known about pathways to treatment of youth with BD and factors that influence the time taken for each stage of these pathways. In this article, we use the conceptual framework by Scott and colleagues called the Model of Pathways to Treatment as a foundation to explore the components of delay in the diagnosis and treatment of youth with BD. The total time from the onset of symptoms until treatment initiation was divided into four sequential intervals; i.e., the Appraisal, the Help-seeking, the Diagnostic and the Pre-treatment intervals and potential disease, patient, and healthcare system/provider factors that influence each interval were identified. This multidimensional conceptual framework can offer a systematic approach to understanding and exploring barriers to early identification and interventions in BD, which is a crucial step in the development of strategies to facilitate prompt diagnosis and treatment. We hope this work contributes to the discussion on delayed diagnosis and treatment of youth with BD and provides a roadmap to inform future research studies and policy decisions.

## 1. Introduction

Bipolar disorder (BD) is a potentially debilitating psychiatric condition characterized by recurrent episodes of depression and (hypo)mania. Statistics Canada data indicates that the lifetime prevalence of BD in Canada is 2.6%, meaning that almost 1 million Canadians are affected (1). BD typically emerges during adolescence and early adulthood and is associated with significant cognitive impairment (2), brain tissue loss (3) and functional disability (4) even in the early stages of the illness. Data from the World Health Organization Global Burden of Disease study ranked BD as the 4th leading cause of disability worldwide among the 10–24 year age group (5). Despite the high prevalence and large disability burden, many studies have shown that BD often goes unrecognized for several years (6, 7). A recently published Canadian multicentre naturalistic study showed that the median delay between the first mood episode and the accurate diagnosis of BD in Canada is 8 years (7). Even more concerning was the median delay of 15 years for pediatric-onset BD. Such prolonged diagnostic delays can result in a subsequent delay in appropriate treatment initiation, which in turn is linked to serious consequences including poor social adjustment (8), disruption of crucial age-specific developmental tasks (9), employment difficulties (10), suboptimal response to mood stabilizing treatment (11), greater severity and frequency of mood episodes (12), higher number of hospitalizations (13), greater risk of developing substance use disorders (14), and elevated risk of suicide (7). In addition, delay in the diagnosis of BD is associated with significantly higher healthcare costs (15) as well as higher indirect costs due to work loss (15, 16). Therefore, accurate identification and appropriate treatment initiation in the earlier stages of BD may lead to improved clinical and functional outcomes and reduction in morbidity in youth with BD as well as cost savings for the healthcare systems.

## 2. Factors affecting delays in the diagnosis and treatment of BD in youth

**Figure 1:**
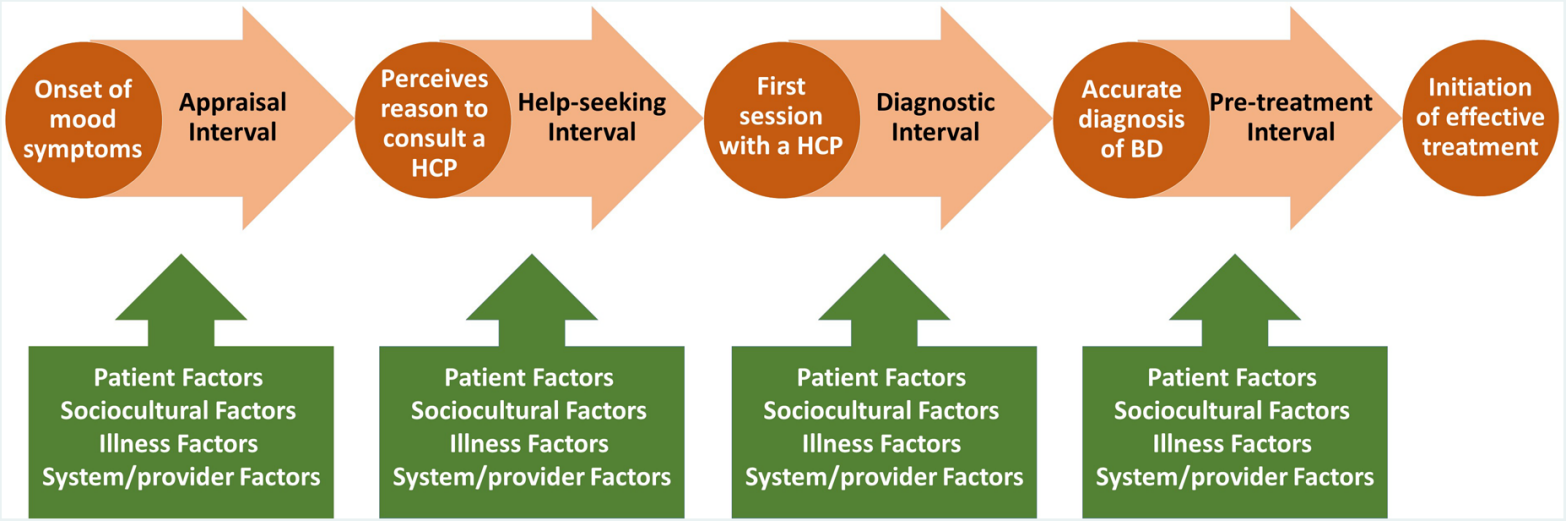
A multidimensional conceptual framework, based on the model of pathways to treatment by Scott et al. (18), to explore potential factors involved in delayed diagnosis and treatment of bipolar disorders in youth. Circles represent key events and arrows represent components of delay.

Previous studies have investigated demographic and clinical factors associated with delays in the diagnosis and treatment of BD. Those would include bipolar disorder subtype (bipolar II disorder), age of onset (younger), polarity of onset (depression), and history of attempted suicide, according to a recently published systematic review (17). However, little is known about pathways to treatment of youth with BD and factors that influence the time taken for each stage of these pathways (i.e., components of delay). A better understanding of these components as well as various factors that can contribute to each component would be a crucial step in the development of strategies to facilitate prompt diagnosis and treatment in youths with BD. To our knowledge, no attempt has been made to analyse components of delay in the diagnosis and treatment of youth with BD. In this article, we use the conceptual framework by Scott and colleagues called the *Model of Pathways to Treatment* (18) as a foundation to explore the components of delay in the diagnosis and treatment of youth with BD and identify potential factors influencing each component. According to this framework, the total time from the onset of symptoms until treatment initiation is divided into four sequential intervals. The Appraisal interval is defined as the time from the onset of mood symptoms to perceiving a reason to discuss such symptoms with a healthcare professional (HCP). The Help-seeking interval describes the time from perceiving a reason to discuss symptoms with a HCP to the first consultation regarding those symptoms. The Diagnostic interval represents the time between the first appointment with a HCP and receiving the formal diagnosis. The Pre-treatment interval describes the time between receiving the formal diagnosis and initiation of treatment (18). Subsequently, various factors contributing to each interval are grouped into three categories: patient factors (that would also include social, cultural and family factors), healthcare provider and system factors and disease factors (Figure 1). In the following sections, we use this categorization to map out potential factors that influence the time taken for each component of delay in the diagnosis and treatment of youth with BD (see summary in Table 1).

**Table 1:** Summary of contributing factors to each component of delay in the diagnosis and treatment of youth with bipolar disorder.

	Appraisal interval	Help-seeking interval	Diagnostic interval	Pre-treatment interval
Disease factors	• Attributing milder symptoms of BD during earlier to developmentally normative mood fluctuations. • Attributing symptoms of BD to comorbid conditions such as substance use disorders.	• Hypomanic symptoms not functionally impairing or perceived as enjoyable. • Depressive symptoms causing low energy, low motivation or negative cognitions about accessing healthcare services.	• Depression being the first or predominant mood polarity. • No reliable method to differentiate bipolar depression from unipolar depression.	• Impaired insight
Patient factors	• Cognitive, behavioural and emotional factors such as the use of heuristics, coping style and self-regulation strategies. • Patient's and family's beliefs towards psychiatric symptoms.	• Lack of knowledge of mental health services, self-stigmatization of mental illness, concerns about confidentiality, comorbidities such as substance use disorder, physical disability. • Resistance to perceived parental control. • Parents’ negative experiences with own BD. • Gateway provider factors (e.g., low mental health literacy, stigma, inadequate financial resources)	• Underreport of manic or hypomanic symptoms during clinical interviews.	• Patient, family and societal attitudes towards psychotropic medications. • Quality of therapeutic alliance with the healthcare provider.
Healthcare provider and system factors	• Underutilization or unawareness of appropriate screening tools for BD in primary care settings.	• Limited access to care providers. • Lack of culturally safe mental health resources.	• Limited time and expertise in primary care settings. • Limited access to specialized mental health services. • Long waiting times to see a youth psychiatrist. • Relying on heuristics for diagnosing as opposed to diagnostic criteria.	• Adherence of healthcare providers to evidence-based treatments. • Affordability and accessibility of recommended treatments.

## 3. The appraisal interval

The time from detection of first mood symptoms to perceiving a reason to discuss symptoms a HCP can be influenced by disease factors, patient factors (including social, cultural and family context), and healthcare/system factors.

### 3.1. Disease factors

According to the “clinical staging model”, BD can evolve through successive stages from an asymptomatic at-risk period to subclinical mood symptoms in youth, followed by full syndromal mood episodes (19, 20). It is reasonable to assume that milder symptoms of BD during earlier stages of the illness can be mistaken for the developmentally normative mood fluctuations seen in adolescents (21).

Another factor contributing to the Appraisal interval is possible misattribution of mood symptoms to other common conditions in this age group such as substance use, which in fact tends to start after the onset of the index mood episode (22, 23) and may represent a self-management strategy to cope with the very symptoms caused by BD (24).

### 3.2. Patient factors

Cognitive, behavioural and emotional factors can influence individuals' response to the newly detected symptoms and how those symptoms are interpreted (25). Those would include patients' attitude towards mood symptoms, and perceived meaning and significance of these symptoms. Patients also use variety of heuristics, colloquially known as “rules of thumb” or “mental shortcuts”, to make sense of their symptoms and form an interpretation (18). Examples of relevant heuristics would include stress-illness heuristics (symptoms that emerge during times of stress are attributed to stress rather than an illness) and severity rule (only symptoms that are persistent or severe and impact day to day functioning requires psychiatric care) (18). Patients' coping style and self-regulation strategies and evaluation of the effectiveness of these strategies to mitigate mood symptoms may impact their decision to seek professional help for their symptoms.

Youth with BD and their families may hold a wide range of beliefs regarding the origins of psychiatric symptoms in general. These beliefs, which include “normalizing attributions” (26) (attributing the symptoms to non-pathological causes such as environmental stressors, or volitional behaviours), supernatural (27), psychosocial and biological attributions (28), can influence the perception of the need to seek professional care.

### 3.3. Healthcare provider and system factors

A recent systematic review and meta-analysis estimated that about 17% of patents who are diagnosed as having a unipolar depression have unrecognized BD (29). This can be at least partly due to the underutilization or unawareness of appropriate screening tools for BD. In fact, it has been shown that screening for BD primary care settings can improve early identification and appropriate treatment of BD (30).

## 4. The help-seeking interval

Several factors can affect the decision to seek professional help for symptoms of BD and therefore contribute to the overall treatment delay.

### 4.1. Disease factors

Depressive episodes have shown to be the predominant and most debilitating mood state among youth with BD (31) whereas periods of mood elevation (especially in the case of hypomania) tend to be shorter, not as functionally debilitating, and even perceived at times as enjoyable (32). This can cause affected youth not to be motivated to seek help during hypomanic episodes. On the other hand, youth may lack energy and motivation to access healthcare services, or may have negative cognitions such as not being deserving of care or being hopeless about getting better during depressive episodes.

### 4.2. Patient factors

Lack of knowledge of available mental health services, self-stigmatization of mental illness, comorbid conditions such as substance use disorder (24), physical disability and concerns about confidentiality may prevent youth from seeking appropriate help. Other patient factors that can influence this interval include individuals' perceived ability to seek help (i.e., self-efficacy) as well as perceived consequences of such help seeking (i.e., outcome expectations) (18, 33). In addition, developmental tasks during adolescence such as the need for independence and individuation from parents can result in resistance to perceived parental control, which can serve a barrier to help-seeking (34). Finally, since about one-third of youth with BD have a biological parent with the condition (35), parents' own experiences with BD may influence help-seeking in youth. More specifically, although having been exposed to symptoms of BD may help with early detection in the appraisal stage, it can also prolong the help-seeking interval especially if it leads to self-stigma or anxiety about the consequences of diagnosis.

The majority of youth help-seeking for emotional distress occur within the informal social network (36), and when it comes to professional help-seeking, youth usually do not seek mental health services on their own (37). Therefore, the role that caregivers, educators and other adults, collectively known as “gateway providers” (37) play in help-seeking in this age group should be recognized. For instance, perceived stigma associated with mental health issues as well as insufficient mental health literacy may prevent some parents and caregivers to seek help for youth who are going through early stages of BD (38). Other potential gateway provider factors include low mental health literacy among educators (39) and caregivers, inadequate financial resources and other psychosocial barriers that may affect caregivers' ability to seek help for the youth.

### 4.3. Healthcare provider and system factors

Potential factors related to healthcare system include limited accessibility to care providers, especially in rural and geographically remote areas, and lack of culturally safe mental health resources for youths and their families.

## 5. The diagnostic interval

The time between the first appointment with a HCP and receiving the diagnosis of BD can be influenced by the following factors.

### 5.1. Disease factors

The official diagnosis of BD requires a history of manic or hypomanic episodes; however, the first (hypo)manic episode is frequently preceded by depressive episodes for several years (23, 40, 41). This can lead to an inevitable delay in the diagnosis of BD and inadequate, ineffective or even harmful interventions. Approximately, a quarter of youth with the initial diagnosis of MDD will transition to BD (42). Cross-sectional studies comparing clinical features of unipolar and bipolar depression in youth have identified features such as high rates of psychiatric co-morbidities, family history of psychiatric illness, higher severity of depression and higher level of functional impairment to be associated with bipolar depression (43). Additionally, prospective studies of adolescents and adults with major depressive disorder have shown that family history of BD, earlier age of onset of depression, presence of psychotic symptoms as well as subthreshold manic symptoms can predict future transitioning to BD (44). However, since no pathognomonic clinical features exist to differentiate bipolar depression from unipolar depression (45), the diagnosis of BD is typically delayed at least until the emergence of first threshold manic or hypomanic episode.

### 5.2. Patient factors

It has been shown that youths with BD tend to underreport their manic or hypomanic symptoms during clinical interviews (46) and therefore, in the absence of reliable collateral information or observable sign of mania during the clinical assessment, they may be diagnosed as having a unipolar depression.

### 5.3. Healthcare provider and system factors

Primary care providers are the first point of clinical contact for many individuals with BD (47) and therefore play a major role in early identification. However, they may not have the time and expertise required to assess youth with BD. In addition, limited access to specialized mental health services for youth with mood disorders and long waiting times to see a youth psychiatrist can contribute to a prolonged diagnostic interval. A recently published systematic review showed that access to early intervention services, except for a minority of individuals with BD, remains limited (17).

The HCP clinical decision-making process can be subject to heuristic biases resulting in diagnostic errors (48). For example, availability bias (tendency to rely on information that comes to mind more readily) can result in consideration of the most plausible diagnosis (18) and outcome bias can cause the healthcare professional to opt for diagnostic decisions that lead to more favorable outcomes or an inadvertent dismissal of BD symptoms. In fact, one study showed that 45% of psychiatrists fail to diagnose bipolar disorder when presented with case vignettes describing an individual in a manic state due to heuristic biases (49).

## 6. The pre-treatment interval

The time between receiving the diagnosis of BD and initiation of effective treatment. During this time, the HCP discusses treatment plans with the youth and their caregivers.

### 6.1. Disease factors

The pre-treatment interval can be impacted by impaired insight (impaired awareness of one's own psychiatric condition, and acceptance of the need for pharmacological or psychological intervention), which is a multidimensional construct and is associated with cognitive and emotional processing and illness characteristics (50). Impaired insight is a common feature of BD; one study showed that the prevalence of impaired insight in symptomatic and remitted adults with BD was 47% and 94% respectively (51). Similarly, another study showed 60% of adults with remitted BD had impaired insight (52).

### 6.2. Patient factors

Patient, family and societal attitudes towards psychotropic medications in general and mood stabilizing medications in particular can influence the pre-treatment interval. For instance, results from the National Stigma Study-Children suggested that approximately half of the responders agreed somewhat or strongly that receiving mental health treatment would “make a child an outsider at school” and “suffer as an adult” (53). Other perceptions that can potentially influence youth attitudes towards mood-stabilizing medications include not wanting to feel different to their friends, feeling pressured by others to stop the medication and not wanting to be reliant on medications (54). Additionally, quality of therapeutic alliance with the HCP (which can also be considered as a provider factor) can influence this interval.

### 6.3. Healthcare provider and system factors

One contributing factor affecting the Pre-treatment interval can include level of adherence of healthcare providers to evidence-based treatments. A recent Canadian study showed that only 59.6 percent of patients with BD received evidence-based treatment recommended by practice guidelines (55). Even the term “pediatric bipolar disorder” and associated interventions remains somewhat controversial among clinicians and academics (56, 57). Other potentially impactful factors include affordability and accessibility of recommended treatments for youths with BD.

## 7. Discussion

The diagnosis and treatment of BD among youth is frequently delayed. Such delay poses a complex public health challenge as it involves different components that can be influenced by a multitude of interconnected disease, patient, and healthcare system factors. The extent to which each of these of factors contribute to the overall duration of undiagnosed and untreated BD in youth remains unknown and therefore warrants further studies. A multidimensional conceptual framework can offer a systematic approach to understanding and exploring barriers to early identification and interventions in BD, which in turn can spur the development of novel interventions and inform policy decisions. In this article, we used the Model of Pathways to Treatment as a foundation to explore potential factors involved in delayed diagnosis and treatment of BD in youth. We must add the caveat that some of the proposed contributing factors were extrapolated from studies of adults with BD or from the broader literature on youth with psychiatric disorders in general.

Fortunately, most of the contributing factors outlined in this article can be potentially amenable to various biomedical, psychosocial or public health policy interventions. As such, a more comprehensive understanding of these interrelated factors can provide countless opportunities for research towards developing innovative early identification strategies across various delay intervals. For instance, the Appraisal interval may be reduced by mental health literacy interventions (58) (patient factor) or through identifying potential biomarkers that would predict future manic or hypomanic episodes in youth who present with—seemingly unipolar—depression (disease factor). Similarly, anti-stigma campaigns (59) may help address patient-related barriers in the Help-seeking interval, which can also be minimized through innovative use of technology to improve access to specialists for youth, families and primary care providers (health-care provider and system factor).

Families, youth, and practitioners who are concerned about the possibility of pediatric BD can consult their local youth mental health services and refer to online resources such as Centre for Youth Bipolar Disorder (https://www.camh.ca/en/science-and-research/institutes-and-centres/centre-for-youth-bipolar-disorder), or Bipolar Disorder in Children and Teens available on the National Institute of Mental Health website (https://www.nimh.nih.gov/health/publications/bipolar-disorder-in-children-and-teens).

We hope this work contributes to the discussion on delayed diagnosis and treatment of youth with BD and provides a roadmap to inform future research studies and policy decisions.

## Data Availability

The original contributions presented in the study are included in the article, further inquiries can be directed to the corresponding author.
